# Pattern of Answer Script Presentation Errors: Teacher and Student Perspectives

**DOI:** 10.7759/cureus.67585

**Published:** 2024-08-23

**Authors:** Asitava Deb Roy, Mala Mukherjee, Anubhav Dwivedi, Deepak Kumar, Shailendra K Yadav, Himel Mondal

**Affiliations:** 1 Health Professions Education, Institute of Health Professions Education, Sri Balaji Vidyapeeth, Puducherry, IND; 2 Pathology, Mata Gujri Memorial Medical College, Kishanganj, IND; 3 Physiology, Autonomous State Medical College Society, Auraiya, IND; 4 Physiology, All India Institute of Medical Sciences, Deoghar, Deoghar, IND

**Keywords:** medical student, essay-type question, examination, answer script writing, diagram, handwriting legibility, teacher perceptions, student perceptions, assessment errors, answer script

## Abstract

Background

Answer script presentation is an effective means of conveying knowledge and understanding. It reflects clarity of thought and organization, which can positively influence scoring. Additionally, well-structured answers reduce the chances of misinterpretation, ensuring that your knowledge is accurately assessed. Despite its importance, there is limited research focusing on the specific errors students make in presenting their answers. Hence, this study explored common errors in answer script presentation from the perspectives of both teachers and students.

Methods

A cross-sectional study was conducted involving 240 students and 50 teachers in July 2024 at Mata Gujri Memorial Medical College, Bihar, India. A questionnaire was developed by a three-member panel of experts in education and assessment to ensure it was comprehensive and relevant to the study's objectives. The questionnaire comprised 12 items rated on a 5-point Likert scale where a higher score indicates higher perceptions of the error. Data were collected from teachers and students using the pre-tested self-administered printed questionnaire. The scores among the different perceived errors were compared by ANOVA and the scores between teachers and students were compared by unpaired t-test.

Results

Students perceived that their highest error was inconsistent handwriting (2.72±1.4), followed by incomplete diagrams (2.52±1.2) and disorganization (2.47±1.17). The error perceived to be least important was incorrect numbering (1.53±0.97), F=12.49, p-value<0.0001. Teachers perceived the error in illegible handwriting (4.36±0.48), followed by lack of emphasis (4.16±0.62) and disorganization (3.94±0.91) as the errors most likely to contribute to poor performance. The error perceived to be least important was inconsistent handwriting (2.4±1.01), F=18.22, p-value<0.0001. When the data were compared between teachers and students, except for inconsistent handwriting, the perceived error score by teachers was higher than the students perceived.

Conclusion

There was a significant disparity between students' and teachers' perceptions of common presentation errors, with teachers consistently rating the severity of errors higher than students. Both groups identified inconsistent handwriting as a prominent error. This underscores the need for better alignment and communication between students and educators regarding the importance of specific aspects of written presentation in assessments.

## Introduction

Answer script presentation plays an important role in academic assessments, where clarity and organization can significantly impact the grading process. Even though students are aware of these errors, it's not unusual for them to submit their scripts with several mistakes that could impact their scores [1,2]. These mistakes not only reflect a lack of preparation but also highlight gaps in educational practices and communication between teachers and students [3].

Although essay-type answer evaluation has subjective components and has some limitations [4,5], it is still considered a necessary component of medical education. Essay-type questions help in assessing students’ in-depth understanding of the topic [6]. In educational settings, answer scripts serve as the primary means through which students demonstrate their knowledge and understanding of a subject. A well-presented answer script allows teachers to accurately assess the student's comprehension, critical thinking, and problem-solving abilities [7,8]. Conversely, poorly presented scripts can obscure the student's knowledge, leading to misinterpretations and potentially unfair grading [9]. Hence, answer script presentation skills are crucial for scoring in examinations.

While several studies have explored effective teaching strategies and assessment methods [10-13], research on the presentation of answer scripts remains relatively scarce. Understanding the common mistakes perceived by both teachers and students can help identify patterns in student errors during examinations. This, in turn, enables educators to develop targeted interventions to improve students' answer script presentation skills.

With this background, this study aimed to examine the perceptions of teachers and students regarding common mistakes in answer script presentations.

## Materials and methods

Study design

This study was a cross-sectional survey to explore the perceptions of teachers and students regarding common mistakes in answer script presentation. The study was conducted at Mata Gujri Memorial Medical College, Bihar, India, in July 2024 after getting approval from the Institutional Ethics Committee of Mata Gujri Memorial Medical College (approval number: MGM/IEC/102/2024).

Questionnaire development

The questionnaire was meticulously developed by a three-member panel of experts in education and assessment to ensure it was comprehensive and relevant to the study's objectives. The development process began with an extensive literature review on answer script presentation, assessment methods, and common student errors. The questionnaire included a part where characteristics of the student or teacher were captured including the age, year of study, study medium in higher secondary from students and age, sex, subject taught, number of copies checked in last year, years of teaching experience, and years of experience as an examiner. The next part contains a set of 12 statements having a five-point Likert response option ranging from "strongly disagree" to "strongly agree." The questionnaire was pilot-tested with a small group of teachers (n=10) and students (n=20).

Participants

The study targeted two primary groups: teachers and students. The teachers included educators involved in grading and assessing answer scripts at various educational levels, while the students consisted of any medical student of the medical college and had written examinations. The study was conducted in a private medical institution situated in the eastern part of India.

Data collection

The questionnaire was printed and distributed among the students and teachers for self-administration. The students were recruited after a one-hour lecture class and teachers were provided the questionnaire in their departments for filling up their responses. All the participants provided written consent for their voluntary participation.

Data analysis

The survey responses were coded as strongly agree=five, agree=four, neutral=three, disagree=two, and strongly disagree=one for quantification. Quantitative data from the survey responses were expressed in mean and standard deviation. The score among the errors was compared by ANOVA with a post-hoc test. The response scores between teachers and students were compared by unpaired t-test. We used GraphPad Prism 9.5.0 (GraphPad Software Inc., United States). A p-value of <0.05 was considered statistically significant.

## Results

A total of 240 students and 50 teachers with a mean age of 22.93±2.26 years and 48.62±8.95 years, respectively, participated in the study. Their characteristics are shown in Table 1.

**Table 1:** Characteristics of students and teachers *: values are expressed either in "number (percentage)" or "mean±standard deviation"

Group	Characteristics	Category	Values*
Students	Age (years)	Male (n=143)	23.04±2.28
		Female (n=97)	22.75±2.22
		Overall (n=240)	22.93±2.26
	Year of study	2nd	83 (34.6)
		3rd	82 (34.2)
		4th	75 (31.2)
	Study medium in higher secondary	English	150 (62.5)
		Vernacular language	90 (37.5)
Teachers	Age (years)	Male (n=28)	51.25±9.62
		Female (n=22)	45.72±6.82
		Overall (n=50)	48.62±8.95
	Subject	Pre-clinical	8 (16)
		Para-clinical	19 (38)
		Clinical	23 (46)
	Teaching experience (years)	Male	13.71±4.60
		Female	12.31±4.37
		Overall	13.1±4.51
	Experience as examiner (years)	Male	9.17±3.89
		Female	8±3.71
		Overall	8.66±3.82
	Number of copies checked in last year	<100	5 (10)
		100-150	10 (20)
		151-200	19 (38)
		201-250	10 (20)
		>250	6 (12)

The student group consisted of 143 males and 97 females. They were distributed across the 2nd, 3rd, and 4th years of study, with 62.5% having studied in English and 37.5% in a vernacular language during higher secondary education. The teacher group comprised 28 males and 22 females, primarily from clinical (46%) and para-clinical (38%) subjects, and had an average teaching experience of 13.1±4.51 years. Most teachers checked between 151 and 200 answer scripts in the last year, indicating substantial experience in examination evaluation.

**Figure 1:**
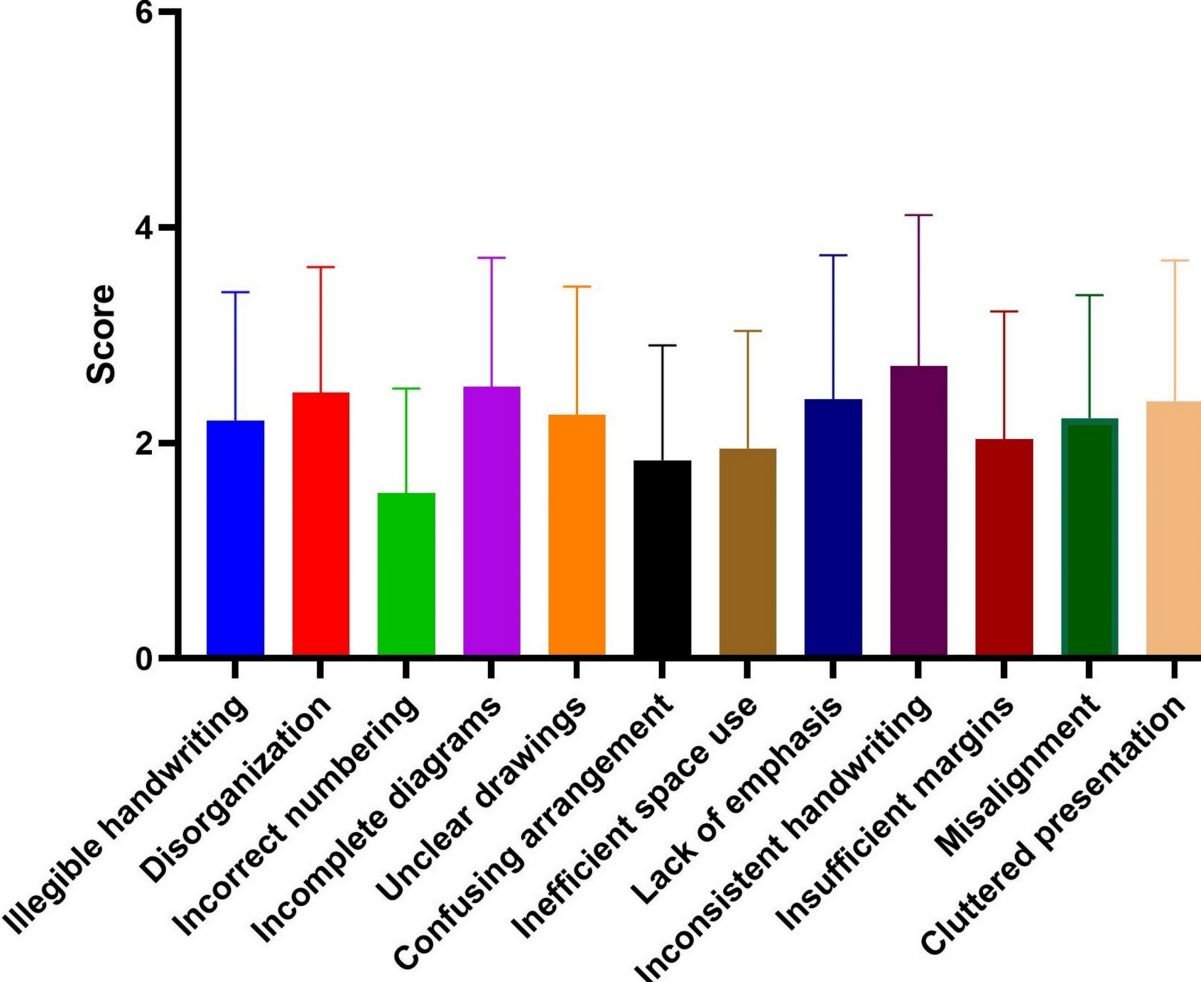
Average scores of students' perceived errors in examination answer scripts

Figure 1 shows the average scores for the attributes perceived by students.

There was a significant difference among the attributes (F=12.49, p-value<0.0001). In the post hoc test, among 66 pairs, 25 showed a statistically significant difference. The highest error was inconsistent handwriting (2.72±1.4), followed by incomplete diagrams (2.52±1.2) and disorganization (2.47±1.17). The least perceived error was incorrect numbering (1.53±0.97).

**Figure 2:**
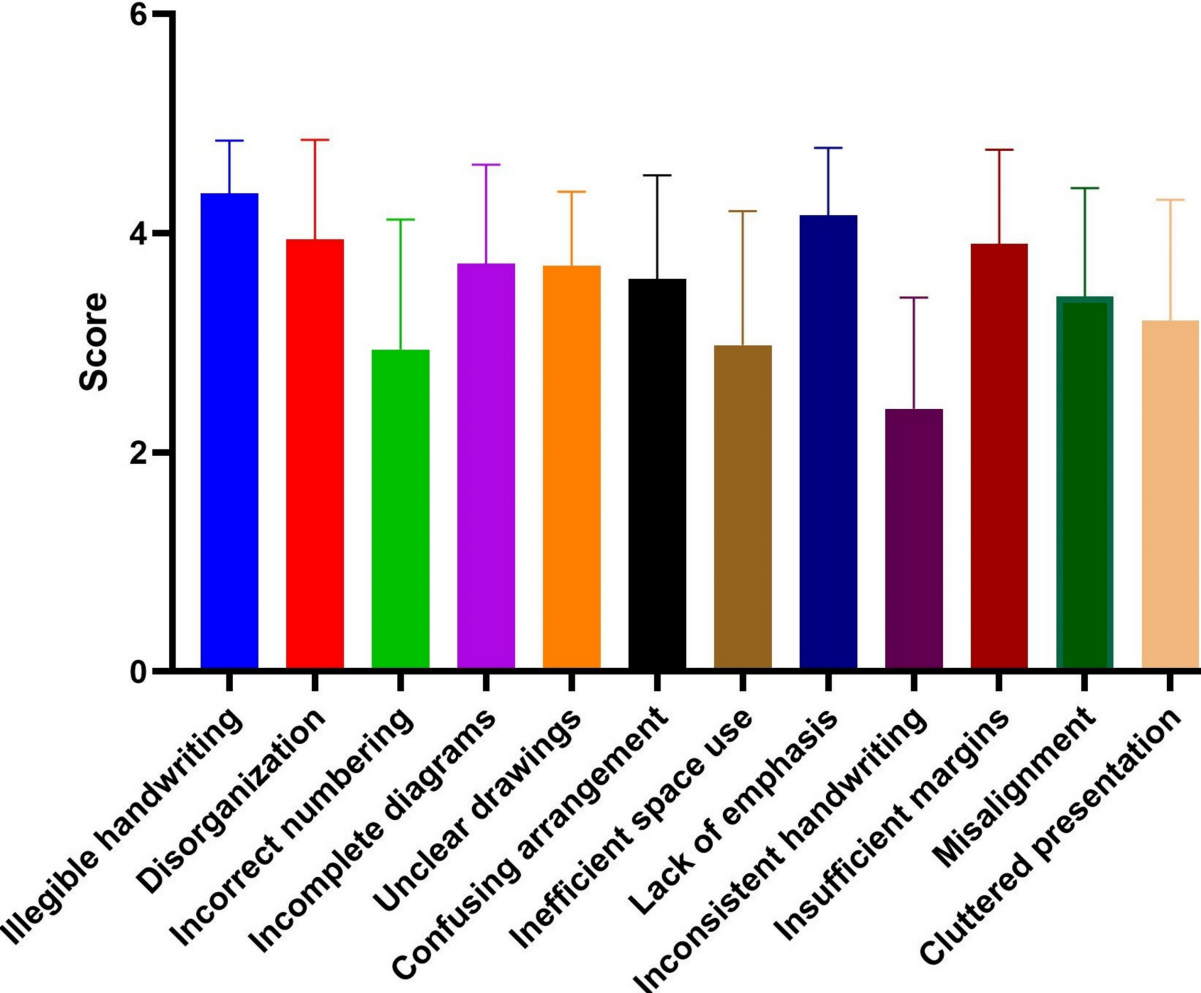
Average scores of teachers' perceived errors in examination answer scripts

Figure 2 shows the average scores for the attributes perceived by teachers.

There was a significant difference among the attributes (F=18.22, p-value<0.0001). In the post hoc test, among 66 pairs, 31 showed a statistically significant difference. Teachers identified the highest error in illegible handwriting (4.36±0.48), followed by lack of emphasis (4.16±0.62) and disorganization (3.94±0.91). The least perceived error was inconsistent handwriting (2.4±1.01).

Table 2 shows the comparative scores between teachers and students.

**Table 2:** Comparative perceived error score between students and teachers The p-value is of unpaired t-test. Values are expressed in “mean±standard deviation.”

Attributes	Students (n=240)	Teachers (n=50)	p-value
Illegible handwriting	2.12±1.19	4.36±0.48	<0.0001
Disorganization	2.47±1.17	3.94±0.91	<0.0001
Incorrect numbering	1.53±0.97	2.94±1.19	<0.0001
Incomplete diagrams	2.52±1.2	3.72±0.9	<0.0001
Unclear drawings	2.26±1.19	3.7±0.68	<0.0001
Confusing arrangement	1.84±1.06	3.58±0.95	<0.0001
Inefficient space use	1.95±1.09	2.98±1.22	<0.0001
Lack of emphasis	2.41±1.33	4.16±0.62	<0.0001
Inconsistent handwriting	2.72±1.4	2.4±1.01	0.06
Insufficient margins	2.04±1.18	3.9±0.86	<0.0001
Misalignment	2.23±1.14	3.42±0.86	<0.0001
Cluttered presentation	2.39±1.3	3.2±1.11	0.004

Only inconsistent handwriting received similar scores from both groups. For all other attributes, teachers perceived more errors in students' scripts than the students themselves perceived.

## Discussion

The study revealed significant discrepancies between students' and teachers' perceptions of common presentation errors in answer scripts. Both groups identified handwriting issues as the most prominent error, but teachers consistently rated the severity of these and other errors higher than students did. This indicates a considerable gap in the perception of answer script presentation quality, with students potentially underestimating the impact of their presentation errors on their assessment outcomes.

Several factors could contribute to these findings. Teachers, with their extensive experience in evaluating a large number of answer scripts, have developed a keen eye for identifying errors that could impede the clarity and readability of student responses. This experience likely makes them more critical of presentation flaws, as they directly impact their ability to assess the content accurately and efficiently [14]. In contrast, students may lack the experience and insight to recognize the full impact of their presentation errors. Their focus might be more on the content of their answers than the presentation, reflecting a gap in training and awareness regarding the importance of a clear and organized answer script presentation [15]. Additionally, the difference in educational backgrounds and levels of emphasis on presentation skills during their prior education may play a role in these varying perceptions. Students educated in vernacular languages might face additional challenges in adapting to the presentation standards expected in higher education conducted in English, further contributing to these discrepancies [16].

The implications of these findings for medical education are significant. Addressing these discrepancies is crucial for improving the clarity and effectiveness of students' answer scripts, ultimately leading to more accurate assessments of their knowledge and understanding. Training programs focused on enhancing students' presentation skills and aligning their perceptions with those of their evaluators could bridge this gap, ensuring a more consistent and fair evaluation process. Incorporating modules on effective answer script presentation into the curriculum, providing detailed feedback on presentation errors, and conducting workshops or seminars led by experienced educators could help students understand and internalize the importance of these skills [17]. Additionally, fostering an environment where students can practice and receive constructive feedback on their presentation skills can build their confidence and competence in this area.

The novelty of this study lies in its exploration of the differing perceptions of presentation errors between students and teachers in a medical education context. This dual-perspective approach provides a comprehensive understanding of the issue, highlighting specific areas where students' and educators' expectations diverge.

Limitations

The limitations include the potential for subjective bias in self-reported data and the limited generalizability due to the specific context and sample size. The use of a self-administered questionnaire might have influenced the responses due to social desirability bias or misinterpretation of the questions. A more comprehensive investigation, encompassing a larger and more diverse sample, the incorporation of objective measures of presentation quality, and an exploration of the impact of specific educational interventions on the reduction of presentation errors, would facilitate a more profound understanding and provide more robust evidence to serve as a foundation for educational practice.

## Conclusions

We found a significant discrepancy between students' and teachers' perceptions of common presentation errors in answer scripts, with teachers consistently identifying a higher severity of errors than students. These findings emphasize the need for enhanced training and awareness among students regarding the importance of clear and organized presentations in their answer scripts. Further research is needed to confirm these findings across different educational contexts and to develop effective strategies for improving answer script presentation.
